# Cancer Care at Times of Crisis and War: The Syrian Example

**DOI:** 10.1200/JGO.2016.006189

**Published:** 2016-08-31

**Authors:** Eman Sahloul, Riad Salem, Wessam Alrez, Tayseer Alkarim, Ammar Sukari, Wasim Maziak, M. Bassel Atassi

**Affiliations:** **Eman Sahloul**, Oakland University William Beaumont School of Medicine, Rochester; **Ammar Sukari**, Wayne State University, Detroit, MI; **Riad Salem**, Northwestern University, Chicago; **M. Bassel Atassi**, Little Company of Mary Hospital/Chicago Medical School, Evergreen Park, IL; **Wessam Alrez**, Alrahma Cancer Center, East Ghuta; **Tayseer Alkarim**, Medical Oncology, Damascus, Syria; and **Wasim Maziak**, Florida International University, Miami, FL.

## Abstract

**Purpose:**

As Syria enters its fifth year of conflict, the number of civilians killed and injured continues to rise sharply. Along with this conflict comes the rapid decline of medical care, specifically cancer care. To determine physician and equipment availability, cancer screening and management, and possible solutions relative to various major cities, a survey was distributed to physicians inside Syria through the help of the humanitarian organization Syrian American Medical Society.

**Methods:**

Online surveys were distributed to both certified oncologists who work in cancer clinics and general physicians who work in rural and mobile clinics inside Syria. Variables assessed were physician specialty, location, population, cost, regional situation (besieged versus government controlled), and resource availability and access. Results were stratified by location and physician specialty.

**Results:**

Survey results revealed a large shortage of specialized physicians and inhibited accessibility to screening and management options in besieged areas compared with government-controlled regions. Physicians within both government-controlled and besieged cities reported limited or no targeted agents, radiation therapy, clinical trials, bone marrow transplantation, positron emission tomography scans, magnetic resonance imaging, and genetic testing.

**Conclusion:**

The Syrian civil war has resulted in suboptimal oncology care in the majority of the region. In consideration of specific deficiencies in cancer care, we recommend several solutions that may better the level of care in Syria: patient education on medical documentation and self-examination; online consultation; and cheap, effective screening methods. The implementation of these recommendations may change the course of cancer care in a country that has deteriorated into the worst humanitarian crisis of the century.

## INTRODUCTION

Patients with cancer who live in developing nations receive suboptimal care. In Syria, cancer care is considered expensive, lacks multidisciplinary teams, and is offered only in limited areas. Newer chemotherapies, targeted therapies, subspecialty oncology services, interventional procedures, microinvasive surgeries, bone marrow transplantation programs, modern imaging, and clinical trials are limited, and health insurance coverage is not available. In 2008, the National Cancer Institute documented 9,468 new cases of cancer in Syria.^1^ In a more focused study, 1,802 new cases of cancer were diagnosed in Aleppo, the second largest governate in Syria (970 men, 832 women). In 1998, an overall crude incidence rate of 72.8 per 100,000 person-years was reported for this population, thereby calling for the importance of established and reliable cancer programs in Syria.^2^ The study found breast and bladder cancers as the leading types, which accounted for one third of all cancer cases. Lung and bladder cancers and leukemias were the most common malignancies among males, whereas breast and cervical cancers and leukemias were the most common among females.^2^

Syria has quickly devolved into the worst humanitarian and medical crisis of the century. By 2016, over 250,000 people have been killed, and over 5 million have fled the violence, torture, and rape and to take refuge in the neighboring countries of Turkey, Jordan, and Lebanon.^3^ In 2015, the United Nations High Commissioner for Refugees registered over 4 million Syrian refugees and estimated 7.6 million internally displaced inside the country.^4^ International medical and humanitarian organizations have not been allowed to enter Syria, which leaves millions to face severe deficiencies in medical care. The United Nations High Commissioner for Refugees reported that over 10.8 million people who live under siege suffer from a shortage of food, shelter, and medical aid. Among these scarcities is cancer care. An estimated 200,000 Syrians have died as a result of chronic diseases such as cancer because of a lack of access to treatment and qualified physicians.^5^

Functioning medical clinics have become rare and expensive. Patients with poor prognoses are turned away due to limited funding; those with better prognoses are turned away due to costly treatments. Since 2013, the majority of health care facilities have been destroyed, which leaves only 47% of Syria’s hospitals and health centers partially functional.^6^ The Global Health Observatory reported that in 2008, 31,000 physicians practiced in Syria, with approximately 15 physicians per 10,000 capita.^7^ Since then, > 15,000 physicians have fled Syria. For example, only one oncologist remains in the large countryside of East Ghouta, with a reported 500,000 inhabitants.^8^ Furthermore, since May 2014, of the 5,000 physicians who practiced in East Aleppo before the conflict, only 40 remain to serve 2.5 million people.^8^

Although cancer incidence in low- and middle-income countries is low, survival rates of those with cancer are worse, with 72% of cancer deaths occurring in low- and middle-income countries.^9^ This occurs not only because patients receive affordable/available treatment rather than optimal treatment but also because of late diagnosis.^9^ With conflict comes a progressive increase in poverty and a decrease in health care; these are now evident in Syrian patients with cancer. Challenges to the problem are evident and include delayed diagnosis, lack of screening programs or continuity of care, treatment interruptions due to displacement and costs, threats to medical personnel, and destruction of infrastructure. The current incidence is difficult to establish inside Syria because a representative sample of the population before and after the conflict are currently unavailable. Incidence may actually seem to decrease during the conflict due to increased undiagnosed cases as a result of the breakdown of diagnostic services; even if carcinogenic materials are used in the conflict, their effect will likely take years before becoming apparent for epidemiologic surveillance to document. This reality guided the context of this study, which is focused on resource availability and general cancer management.

We investigated how conflict influences availability of, access to, and quality of cancer care in Syria. We have identified the most common cancers, explored existing diagnostic tools and treatment options, identified preventive screening measures and follow-up efforts, compared government-controlled areas to besieged areas, compared general clinics to specialized cancer clinics, and proposed plausible solutions from an oncologic perspective.

## METHODS

This is a cross-sectional study among several physicians who practice in various locations within Syria. Data were collected through self-administered, online, anonymous, translated Google questionnaires from certified oncologists and surgeons who work in cancer clinics inside besieged and government-controlled major countrysides of Syria and general physicians who work in various locations in Syria, including rural and mobile clinics. Clinics were selected from the Syrian American Medical Society database.

A 30-question survey was generated and distributed to general practicing physicians who work in rural and mobile clinics inside Syria (Data Supplement). A 70-question Web-based survey was distributed to Syrian oncology practitioners in seven major Syrian countrysides: four government-controlled major cities (Damascus, Latakia, Homs, and West Aleppo) and three besieged large cities (East Ghouta, East Aleppo, and Idlib; Data Supplement). Analyses of Aleppo were separated into east and west because the armed conflict has divided the city into east, which is currently under opposition control, and west, which remains under government control.^10^

Because of the small participant pool, the information gathered is considered pilot data for future cancer research in Syria. Variables assessed were physician specialty, location, population, cost, regional situation (ie, besieged versus government controlled), resource access, and resource availability.

## RESULTS

### Cancer Clinics Inside Syria

#### Physician availability.

The survey results indicate that 20 oncologists work in the capital city of Damascus. In the neighboring besieged East Ghouta countryside, one oncology-certified physician is reported. In East Aleppo, where 500,000 people reside, no treating oncologists are available, and thus, all patients with cancer often are transferred to Turkey for treatment. In contrast, four certified oncologists are available in government-controlled West Aleppo, but access to them from besieged East Aleppo is limited and dangerous. An oncology pharmacist is available only in the cancer clinic in Homs, although all participants denied having one available. In Idlib, the cancer clinic reportedly has only two general physicians and more than 10 midwives at any given time. In the entire city, there are no certified oncologists and only one student who at the time had 1 year of training in oncology. Furthermore, all participants noted that the cancer centers take care of both adults and children. Results based on city/countryside and population are summarized in Table 1; populations are as reported by survey participants in the area.

**Table 1:**
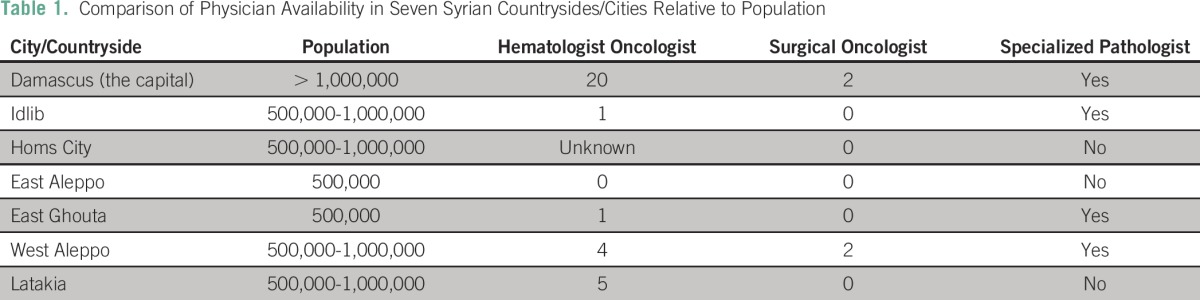
Comparison of Physician Availability in Seven Syrian Countrysides/Cities Relative to Population

#### Resource and treatment availability.

Basic diagnostic tools are available in all clinics, including laboratory blood tests, biopsies, and basic imaging studies. Although magnetic resonance imaging is available in major cities, it is limited in besieged areas. Radiation therapy is only available in Damascus and Latakia, which restricts access to many patients who live under siege or are required to cross military checkpoints. Positron emission tomography scanners are only available in Damascus and require self-pay. Advanced services such as interventional radiology and genetic testing are not available. More-advanced options such as bone marrow transplantations and clinical trials are also not available. Patients who can afford the costs are referred to neighboring countries. However, most patients do not have the capability of traveling or paying for such expensive treatments, which leaves this population of patients with cancer without treatment opportunity.

Chemotherapy was reported to be available in all cities, although the medications are extremely difficult to import into besieged areas. Survey participants from government-controlled cities listed trastuzumab, rituximab, sorafenib, and imatinib as available treatment options; besieged areas did not have access to targeted therapies, except East Ghouta where the participant reported the availability of imatinib. With regard to mortality, only Homs and East Ghouta responded. The participant from Homs stated that 30 people died as a result of cancer in 2013, and 22 died in 2014. In East Ghouta, 20 people who died as a result of cancer in 2013, and 41 died in 2014. Breast cancer was the most common malignancy reported in this study, with all survey participants ranking it first among other cancers. Hematologic, colon, and lung cancers were the next most common, respectively.

#### Patient screening and follow-up.

Only Damascus offered preventive screening measures such as mammograms, colonoscopies, and Papanicolaou smears. Only two clinics reported an established follow-up system after treatment completion. Only Homs and East Ghouta used computerized documentation systems; all others reported having to use written documentation.

#### Online consultation.

Survey participants had different opinions on the value of Internet and online consultation, which they found was of limited value to physicians working in hematology/oncology at cancer clinics inside Syria, except East Aleppo. Their reasons indicated include limited Internet access and sufficient knowledge on cancer cases, whereas general physicians found online consultation beneficial for managing patients with cancer. Table 2 summarizes the resource availability in Syria and compares it to that in US cancer clinics.

**Table 2:**
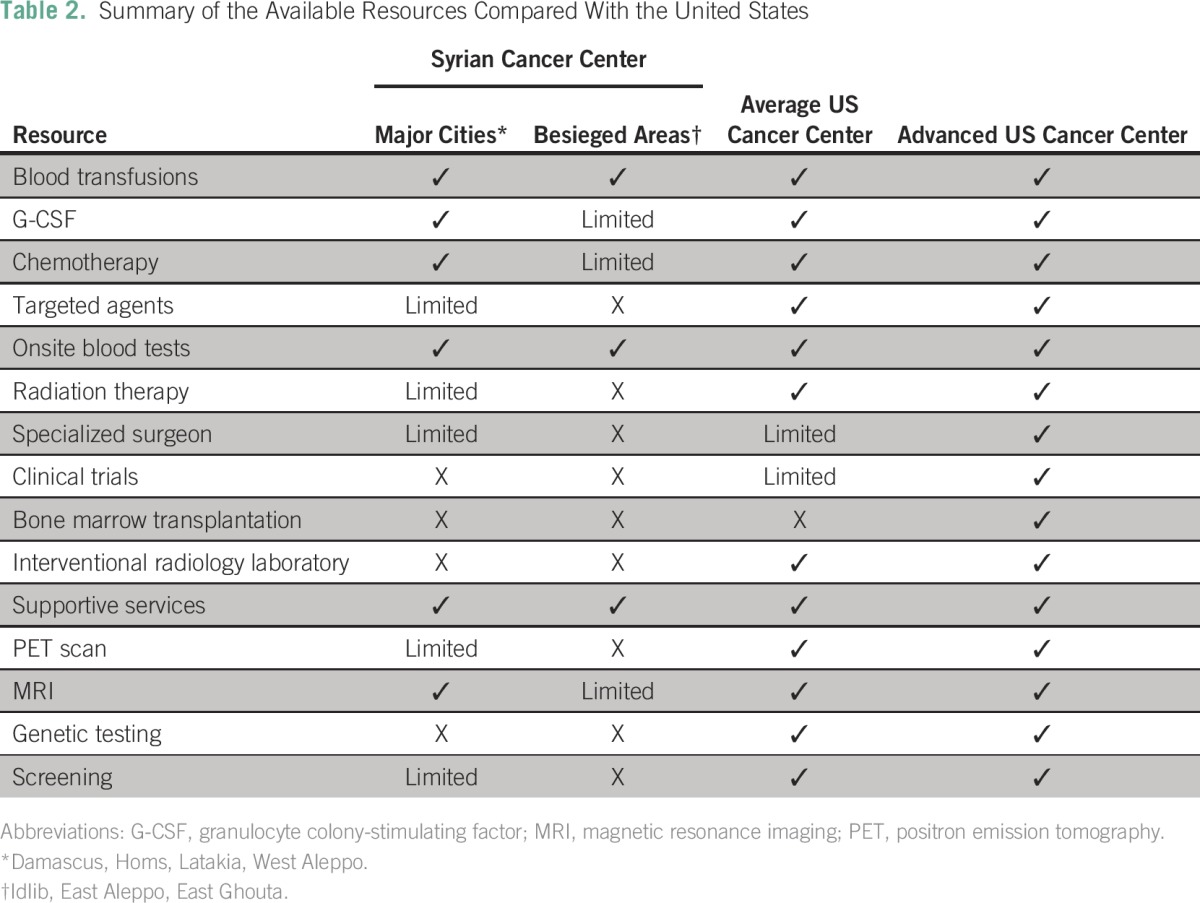
Summary of the Available Resources Compared With the United States

### General Medical Clinics Inside Syria

The survey targeted three areas of northern Syria: East Aleppo, Idlib, and Hama. These areas were chosen because they are the largest cities outside government control but still under conflict. Three survey participants were general surgeons, one was an orthopedic surgeon, and three were general practitioners.

#### Physician availability.

Participants from Hama and East Aleppo admitted that mistakes in diagnosis were made as a result of poor resource availability and no specialized physicians. An American oncologist who volunteered in one of the clinics in Idlib explained that the clinic had only two general physicians and 10 nurses. The physician also reported that in the entire city, there were no certified oncologists and only one student who had 1 year of training in oncology. Physician availability in other areas was limited to general practitioners.

#### Resource and treatment availability.

Survey participants from East Aleppo and Idlib stated that computed tomography scans are limited to certain locations, whereas in Hama, they are available. Of the three countrysides, only Idlib confirmed the availability of chemotherapy drugs. All participants stated that no government-run hospitals existed in the areas that were safe to travel to and no non-government organization (NGO)–based clinics to assist with patients with cancer; consequently, these patients are referred to Turkish cancer centers. All participants noted that patients often leave Syria for cancer treatment in neighboring countries but return because of the high treatment costs.

#### Patient screening and follow-up.

All participants stated that no screening protocols, colonoscopies, and mammograms are available. If physicians are suspicious of possible cancer because of indicators such as unintentional weight loss or a visible mass, they either perform a biopsy on the masses or refer patients to Turkey for further investigation. Follow-ups are rare due to limited resources and no system for keeping medical records in the clinics. Only participants in East Aleppo confirmed that approximately 10 to 30 patients with cancer came to their general clinics in 2014 and 12 to 35 in 2015, whereas participants from Hama and Idlib had no records of how many patients with cancer had come in.

### Cost of Treatment in Syria

Besides limited resources, cost of cancer care is prohibitive to attaining treatment. The survey results suggest that monthly cancer costs range from $100 to $1,000; thus, centers can only buy limited amounts and cheaper alternatives of drugs. Although these numbers seem reasonable for cancer treatment, a 2013 article in *Al-Akhbar* reported that the median salary of working Syrians has plummeted to $150 per month, whereas the cost of basic goods have increased 300%.^11,12^ One chemotherapy course costs roughly the same as the annual per capita, translating to American patients paying $47,000 for that same course. Although besieged participants are supported by NGOs, funding is insufficient. In Damascus and Homs, there are no large NGOs, and patients pay out of pocket. There are two treatment centers in Damascus that patients are referred to for free; however, this government option is not available for people in political opposition areas or under siege.

## DISCUSSION

### Cancer Care in a War Zone and Besieged Cities

#### The most common malignancy.

Breast cancer is now the most commonly diagnosed cancer in women and the second leading cause of cancer death among women^13^; it is also the most common malignancy among Syrians as reported by survey participants. In accordance with the study results, multiple essential diagnostic and treatment options were found inadequate or unavailable. Early detection is the single most important factor in surviving breast cancer because the less advanced the disease, the easier and less costly it is to treat.^13^ According to the American Cancer Society, 98% of women who receive their diagnosis and are treated early are still alive 5 years later compared with 84% of women whose disease has spread to the lymph nodes before treatment starts and 28% whose cancer has spread to distant organs.^13^ As indicated by the study results, magnetic resonance imaging was either unavailable or limited to certain regions and, thus, could not be used to supplement traditional diagnostic procedures in identifying locoregional extensions in younger patients.^14^ Although radiotherapy and lumpectomy have equal benefits to total mastectomy in early stages of the disease, the lack of radiation therapy and specialized breast surgeons may translate to an overly aggressive surgery.^15,16^ Targeted agents, such as trastuzumab, are of no benefit to Syrian patients with human epidermal growth factor receptor 2 amplifications because of limited availability and expense^17^; only survey participants from East Ghouta, Homs, and Damascus reported having access to trastuzumab. Furthermore, oncotype diagnostic testing is not available to guide treatment in patients with early-stage breast cancer who could benefit from or avoid chemotherapy.^18^

### Challenges

#### Deficiencies in the standard of care and first-line treatment.

Aside from breast cancer management, deficiency of first-line treatment options for many other malignancies exist in Syria. As mentioned previously, targeted and newer chemotherapeutic agents are scarce and thus of insufficient benefit for patients with any kind of cancer. The benefits of radiation therapy for the management of head and neck^19^ and stage III lung cancers^20^; adjuvant therapy for breast,^16^ anal,^21^ and prostate cancers^22^; and neoadjuvant therapy for gastroesophageal and rectal cancers^23^ and other malignancies are not available in any of the Syrian countrysides besides Damascus and Latakia. Once again, this shortage causes a significant loss of therapeutic and survival benefits. In addition, autologous stem cell transplantation for multiple myelomas^24^ as well as allogeneic stem cell transplantation for acute leukemias,^25^ eligible myelodysplastic syndromes,^26^ and aplastic anemias^27^ are not available as treatment options in Syria.

#### Lack of specialized physicians.

The Radiation Research Program at the National Cancer Institute recommends that each city must have at least two surgical oncologists, two radiation oncologists, and two hematologist oncologists.^1^ As shown in Table 3, Damascus was the only city that met the National Cancer Institute recommendations, whereas all other cities had either zero or one of the specialized physicians listed.

**Table 3:**
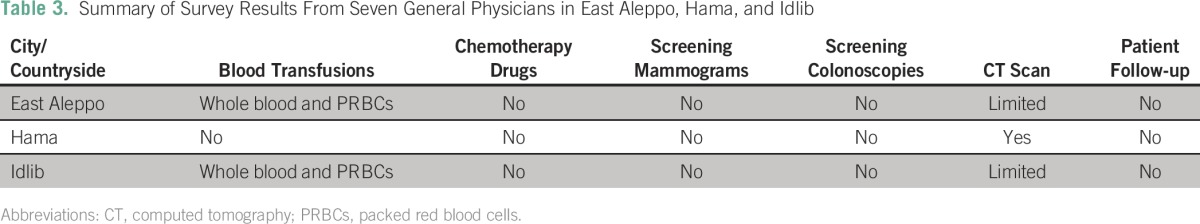
Summary of Survey Results From Seven General Physicians in East Aleppo, Hama, and Idlib

#### Limited screening.

More than one third of cancers are preventable, and another one third are potentially curable provided they are detected early.^28,29^ In Syria, however, the technology and experience for screening are lacking, which calls for a reconstruction in the country’s approach to early detection.

#### Limited follow-up.

As observed in the survey results, patient follow-up is inadequate in Syria for two chief reasons: Volunteer physicians treat patients for limited amounts of time, and computer/chart systems have not been established. Unless patients personally maintain a meticulous and accurate medical history, no records are kept for blood test and biopsy results and drug treatments and dosages. WHO recommends collection of the following information whenever possible^30^: demographic and socioeconomic data (eg, patient name [identification], sex, date of birth, place of birth), financial data related to the payment of fees for medical services and hospital accommodation, and clinical patient data.

### Plausible Solutions

As demonstrated in the study results, a wide discrepancy exists among Syrian cities and countrysides with regard to basic diagnostics, specialized physicians, radiation therapy, chemotherapeutic agents, and even a safe commute. Although Damascus and Latakia have suboptimal cancer management options, they remain acceptable compared with other areas, which essentially includes the rest of Syria. Thus, solutions to current cancer care in Syria depends on geographic location and political affiliation. Opposition areas, for instance, not only face the worst fortune in cancer care but also must survive the least livable conditions. If an internationally protected zone is established, a specialized cancer center that receives referrals from other areas of the country could provide basic diagnostics and treatment options, radiation therapy, a diagnostic pathology laboratory, and chemotherapeutic agents to solve the cancer epidemic in Syria.

#### Online consultation.

Physicians who work in general medical clinics inside Syria found online consultation and Internet access beneficial to the treatment of patients with cancer. Such opportunities for telehealth and electronic medical consultation will give Syrian and volunteer physicians broader treatment options and allow for accurate diagnoses.^31^ The Syrian American Medical Society has developed telemedicine strategies to support physicians through video cameras, Skype, and satellite Internet to ensure high-quality care to patients with trauma injuries and chronic disease.^32^ The current survey has helped to solidify the significance of maintaining a connection with physicians inside areas where medical access and consultation are limited.

#### Patient education.

In an environment where tangible resources and therapies are not available, patients must participate in maintaining their own health and remain aware of their exposure to possible risk factors. In breast cancer, for instance, the development of culturally sensitive local education programs that teach women the significance of self-examination can result in a tide-changing decrease in mortality rates.^33^ Such an initiative can function as a mode of early detection and thus reduce the typical presentation of an advanced disease stage in disadvantaged areas.

### Conclusion

A structured initiative that focuses on patient/physician education, equipment, and availability of specialized physicians and that creates referral facilities in safe zones can positively change the course of cancer care inside Syria. Long-term adjustments are needed to ensure the sustainability of medical care. Such actions may alter the disastrous outcomes in a country that faces the worst medical and humanitarian crisis of the century.
